# Clostridium difficile Infection Complicated by Transformation of Myelodysplastic Syndrome to Acute Myeloid Leukemia With Leukostasis and Sweet’s Syndrome

**DOI:** 10.7759/cureus.19085

**Published:** 2021-10-27

**Authors:** Ian Landry, Mallorie Vest, Anthony Williams

**Affiliations:** 1 Medicine, Icahn School of Medicine at Mount Sinai, NYC Health + Hospitals/Queens, Queens, USA; 2 Internal Medicine, Icahn School of Medicine at Mount Sinai, NYC Health + Hospitals/Queens, Queens, USA

**Keywords:** sweet syndrome, leukostasis, leukemic blast transformation, acute myeloid leukemia, myelodysplasia

## Abstract

Myelodysplastic syndrome (MDS) is a premalignant condition characterized by clonal proliferation and ineffective hematopoiesis. The subtype of MDS associated with deletion in the long arm of chromosome 5 is generally associated with older females and carries a good prognosis as it rarely transforms to acute myeloid leukemia. The mechanisms of leukemic transformation are still poorly understood and likely involve a variety of somatic mutations and epigenetic modifications. We present the case of a 70-year-old female with known MDS with deletion 5(q) who presented with anemia, thrombocytopenia, and guaiac positive stool who was subsequently found to be positive for *Clostridium difficile *infection. During the course of her treatment, she developed significant leukocytosis, splenic infarction, and acute hypoxic respiratory failure requiring high flow nasal cannula. Flow cytometry returned positive for increased blasts of more than 30%. She was transferred to a tertiary care facility for cytoreductive therapy and developed leukostasis and Sweet's syndrome.

## Introduction

Myelodysplastic syndrome (MDS) is a complex entity characterized by ineffective hematopoiesis due to aberrant clonal proliferation and increased apoptosis [1]. The prevalence of MDS is estimated to be approximately 60,000 - 170,000 persons in the United States [2]. While often considered a disease of the elderly population, certain groups such as patients with Down's syndrome, Fanconi anemia, or previous treatment with chemoradiotherapy have been shown to have increased risk [1]. While inherently problematic due to pancytopenia, a significant threat of this syndrome is its progressive transformation to acute myelogenous leukemia (AML). This transformation occurs in approximately one in three patients with MDS and may ultimately be fatal [3]. 

Recent updates in MDS classification were released in 2016 by the World Health Organization (WHO). This classification more accurately characterizes MDS into six separate entities, each with different frequencies of transformation to acute leukemia. The six types are MDS with multilineage dysplasia (MDS-MLD), MDS with single lineage dysplasia (MDS-SLD), MDS with ring sideroblasts (MDS-RS), MDS with excess blasts (MDS-EB), MDS with isolated del(5q), and MDS unclassifiable (MDS-U). The most common type of MDS is that of MDS-MLD, where at least two or three cell types show at least 10% dysplasia. MDS-EB is most likely to transform into AML and represents approximately 25% of MDS. MDS-EB is further subdivided based on percentage of blasts in the bone marrow (type 1, 5-9% of the marrow; type 2, 10-19% of the marrow). MDS with isolated del(5q) is a type of MDS that occurs more often in older women and tends to have a good prognosis with infrequent transformation to AML. MDS-U, MDS-SLD, and MDS-RS are uncommon types that rarely, if ever, progress to acute leukemia.

## Case presentation

A 70-year-old female with a past medical history of myelodysplastic syndrome with 5(q) deletion which was diagnosed three years prior to presentation and complicated by pancytopenia which eventually became transfusion dependent, a remote history of uterine cancer status post hysterectomy and radiotherapy, a history of Sweet's syndrome previously treated with colchicine, essential hypertension, rectal hemorrhoids, osteoporosis and depression presented with generalized fatigue, dizziness upon changing position, nausea, and loss of appetite for three days prior to presentation. The patient denied any melena, hematochezia, falls, trauma, loss of consciousness, chest pain, or dyspnea. On presentation, her heart rate was 95 bpm, she was normotensive at 113/66 mm Hg, afebrile, and maintained her saturation at 98% on room air. Overall, she was well-appearing without significant distress. Her cardiopulmonary examination was without rales or tachypnea. There was mild tenderness to deep palpation in the left lower quadrant without evidence of hepatosplenomegaly. Her examination was negative for any petechiae or rash. Initial lab work showed hemoglobin 6.9 g/dL (baseline around 7-9 g/dL) and platelets 15,000/mcl (baseline around 30,000-50,000/mcl) with normal total leukocyte count of 8,280/mcl.

**Figure 1:**
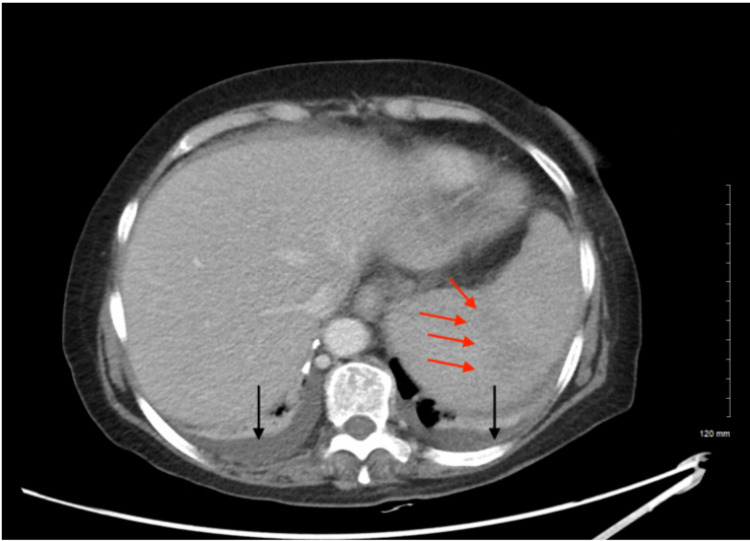
CT of the Abdomen/Pelvis with contrast showing splenomegaly with subtle wedge-shaped low density in the superior spleen (red arrows) concerning for a splenic infarction. Note small bilateral pleural effusions (black arrows).

The patient was admitted with the impression of symptomatic anemia of blood loss requiring transfusion and was given one unit of packed red cells and one unit of platelets with improvement to 7.7 g/dL and 36,000/mcL, respectively. On the second day of her admission, she was noted to have six loose, non-bloody bowel movements. The workup of her diarrhea returned significant for *Clostridium difficile* toxin and oral vancomycin 125 mg every six hours was started. The patient developed worsening leukocytosis of 16,700/mcL and was started on Flagyl 500 mg daily. Abdominal imaging was significant for splenomegaly with a subtle wedge-shaped low density in the superior spleen worrisome for splenic infarction (Figure 1). Over the course of two days, her leukocytosis more than doubled to 36,700/mcL with elevated absolute neutrophilia. During this time, her diarrhea did not improve and infectious disease consultation recommended switching antibiotics to vancomycin 500 mg every six hours. However, the patient began to develop severe abdominal pain, desaturated to 86% on room air and required 3 liters of nasal cannula. D-dimer levels were elevated at 511 ng/mL (ref: 0-243 ng/mL) with normal fibrinogen 211 mg/dL (ref: 200-393 mg/dL) and international normalized ratio (INR) 1.7. Angiogram of the chest and doppler of the lower extremities were both negative for venous thromboembolism. Hematology consultation recommended flow cytometry to examine peripheral blood and characterize leukocytosis. Her worsening clinical status prompted evaluation by a tertiary care facility and she was subsequently transferred for further diagnosis and management. 

**Figure 2:**
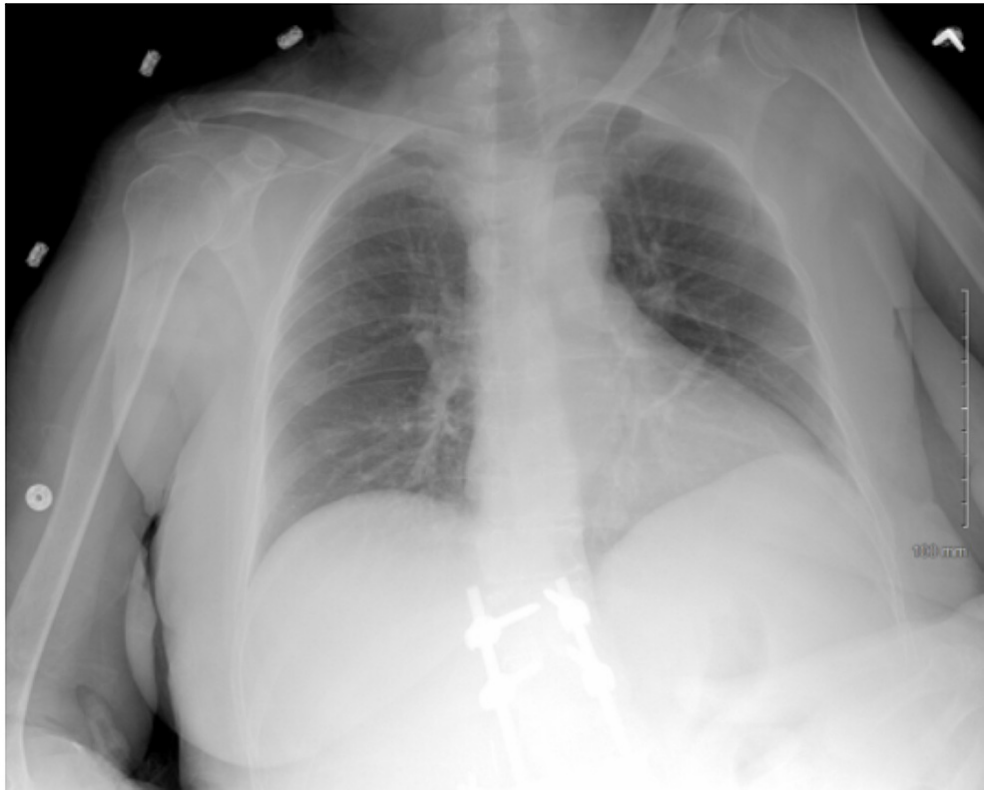
Chest XR from initial presentation

**Figure 3:**
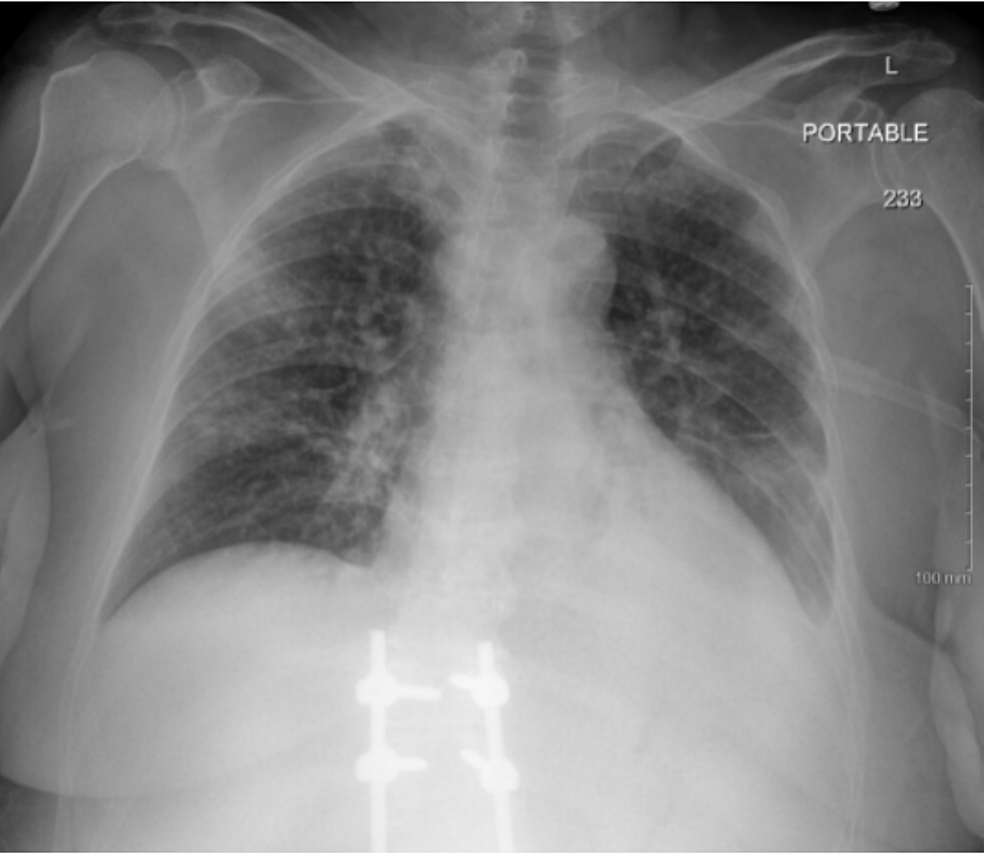
Chest X-ray on presentation to tertiary care center.

**Figure 4:**
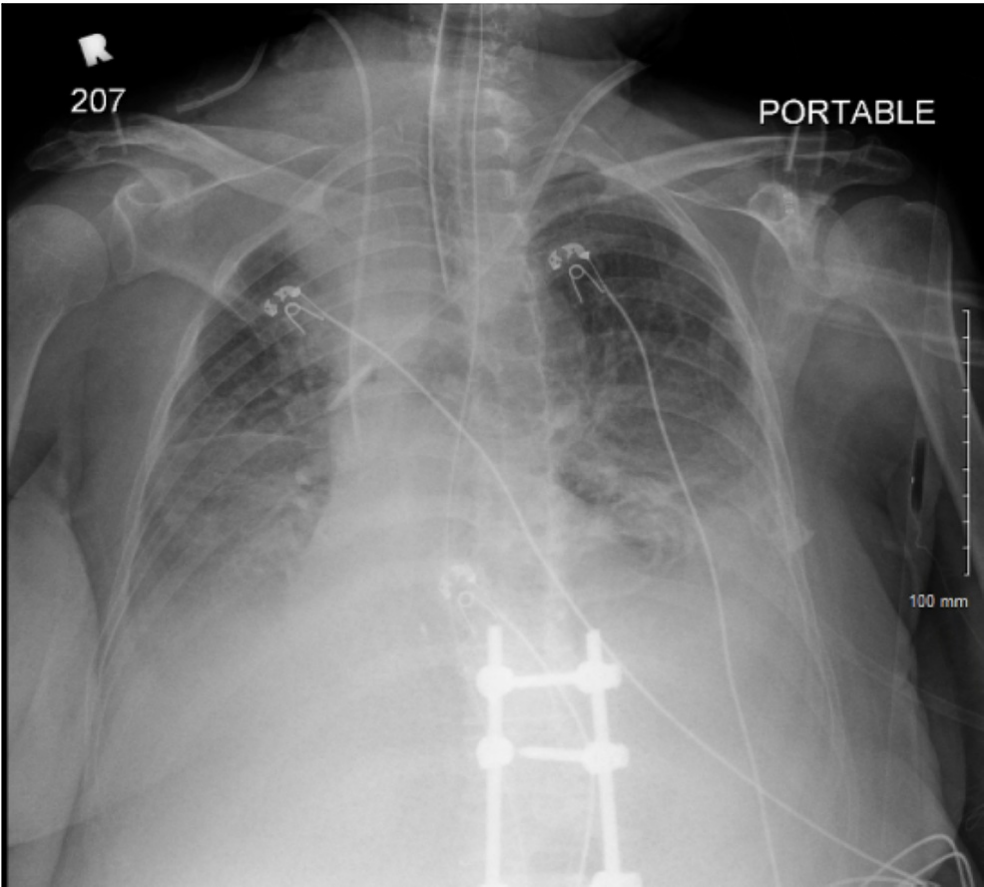
Final Chest X-Ray two days prior to expiration.

Upon presentation to the tertiary center, her total leukocyte count exponentially rose to 99,400/mcL with hemoglobin 6.0 g/dL and platelets 24,000/mcL. Radiograph of her chest (Figures 2-4) showed an evolution of multifocal opacities throughout the lungs with definite consolidation in the left lower lobe and small bilateral pleural effusions. The patient was started on Hydrea 2 grams every eight hours, allopurinol 300 mg every eight hours and covered with vancomycin and cefepime for healthcare-associated pneumonia. Outside records indicated that the patient had undergone a repeat bone marrow biopsy two weeks prior to initial presentation with an increase in cluster of differentiation-34 (CD-34) blast cells ranging from 5% to up to 8% in some areas. These findings were consistent with MDS with excess blasts and the outpatient plan was to begin treatment with venetoclax. Flow cytometry from her initial presentation returned with increased blasts over 30%, including myeloblasts, monoblasts, and promonocytes. Fluorescent-in-situ-hybridization (FISH) analysis of the chromosomal abnormalities returned significant for monosomy of chromosome 7, rearrangements in the short arm of chromosome 12, and isochromosome 18q. These findings confirmed a diagnosis of transformation to acute myeloid leukemia. Her work of breathing increased and her oxygen saturations continued to decline, therefore, the patient was upgraded to the intensive care unit and started on high-flow nasal cannula at 30 L/min. Treatment with decitabine 29.6 mg for five days was started with drastic improvement in her leukocytosis from 99,000/mcL to 10,000/mcL.

**Figure 5:**
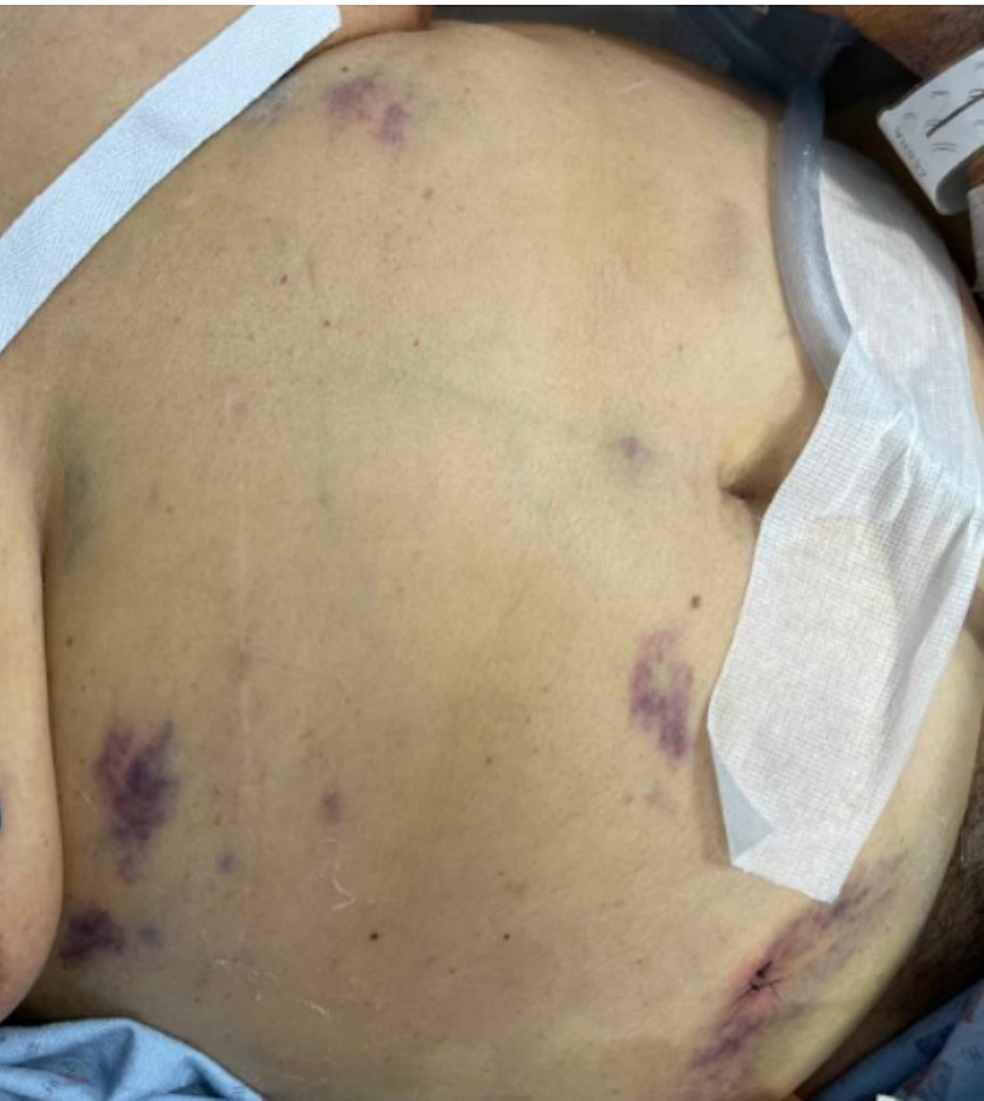
Photo of abdominal lesions.

**Figure 6:**
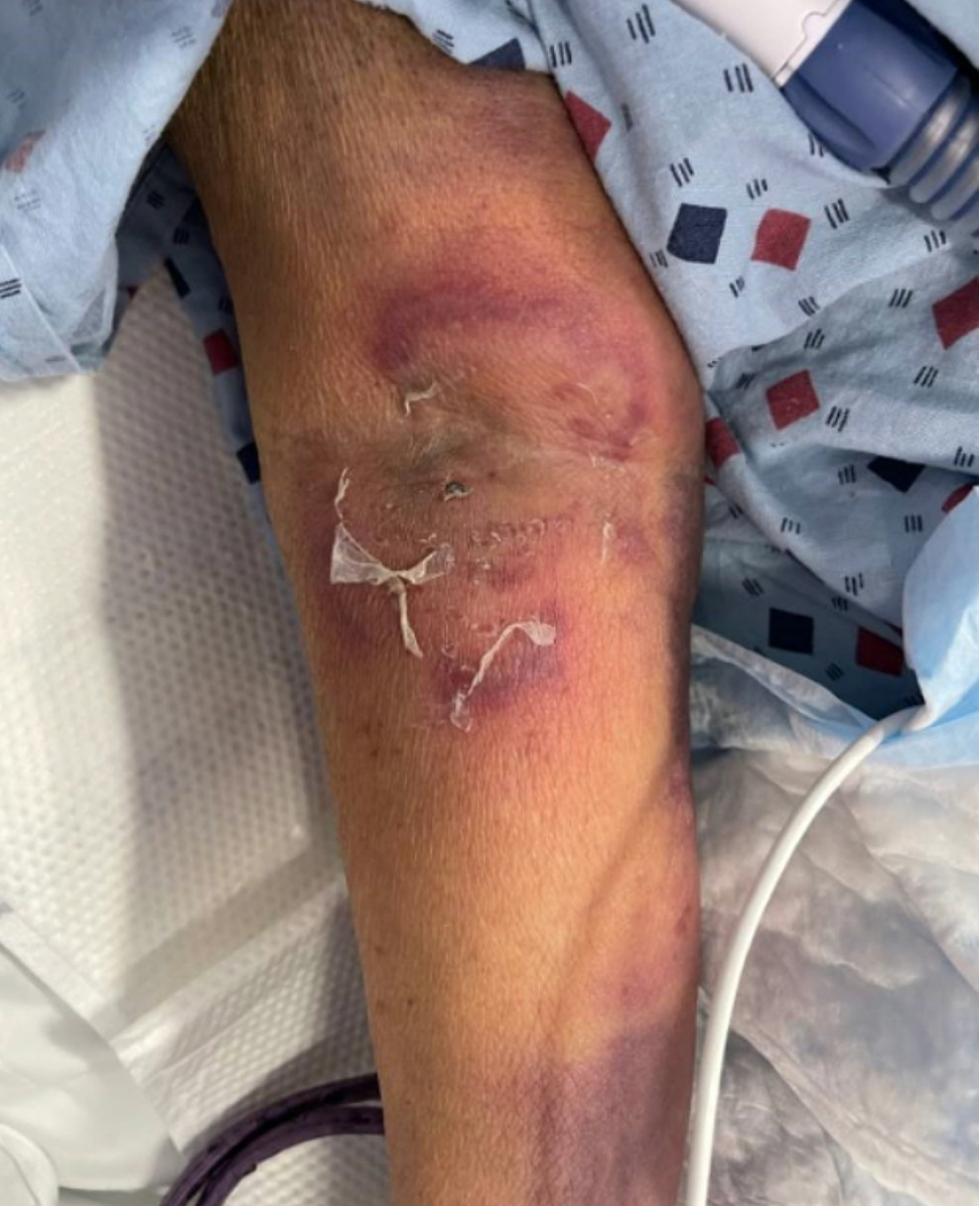
Photo of right antecubital and forearm lesions.

**Figure 7:**
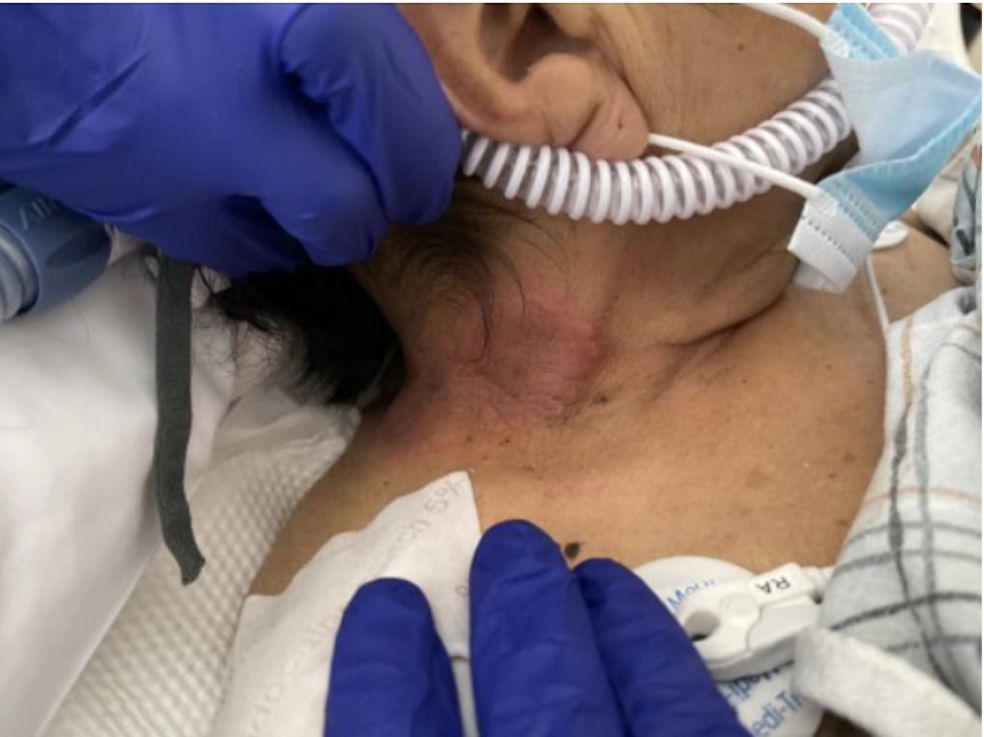
Photo of right sided neck lesion.

As her oxygenation improved, she was downgraded to the step-down unit where she developed fever and a swollen right upper extremity. Antibiotics were continued with the addition of fluconazole 400 mg and acyclovir 400 every 12 hours. Upper extremity duplex was positive for superficial venous thrombosis of the right cephalic vein, but no evidence of deep venous thrombosis. Physical exam was significant for a painful, pruritic rash on the right side of her neck which prompted Dermatology consultation. The patient’s physical examination was significant for an erythematous annular plaque with underlying induration on the right side of her neck and several purple macular lesions on the trunk and lower extremities (Figures 5-7). Colchicine was resumed for the treatment of likely Sweet's syndrome and punch biopsy of the lower extremity lesion showed ecchymoses without evidence of leukemia cutis. Dermatology's impression was that right neck and right arm lesions were clinically consistent with Sweet's syndrome and treatment with methylprednisolone 60 mg every 12 hours was started. 

**Figure 8:**
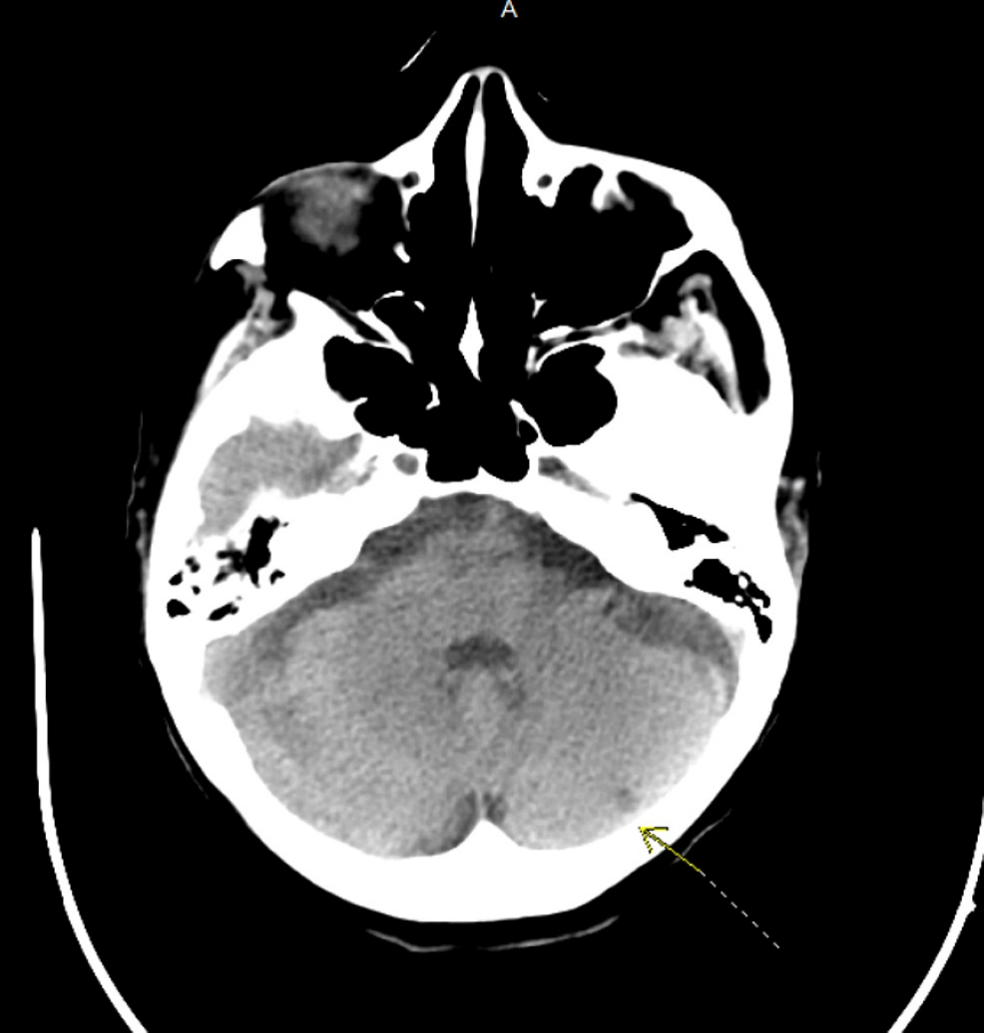
CT head without contrast showing a thin, predominantly low-density sub-acute to chronic fluid collection (yellow arrow) along the inferior left frontal lobe with mild mass effect on adjacent parenchymal structures

During the course of her treatment, the patient developed seizure activity and became hypoxic to oxygen saturation in the 80s while on high flow nasal cannula. Repeat imaging of the chest showed diffuse bilateral opacities and her laboratory findings were significant for lactate of 8 mmol/L, absolute neutrophil count (ANC) of 100/mcL, platelets 38,000/mcL, and hemoglobin 4 g/dL. Her antibiotics were upgraded due to neutropenia and she was evaluated with non-contrast head CT (Figure 8) which showed subacute to chronic fluid collection along the inferior left frontal lobe with a small acute component and mild mass effect on adjacent parenchyma. The patient was transferred to the intensive care unit and started on vasopressors for worsening hypotension. Glucocorticoids were started for Sweet's syndrome and pneumonitis. Over the next several days, renal function declined, the patient remained febrile with worsening pneumonia despite broad-spectrum antibiotics, and the family decided that chest compressions were not within goals of care. The patient was subsequently made do-not-resuscitate (DNR) and expired later that evening.

## Discussion

Deletion of the long arm of chromosome 5 (del (5q)) remains one of the most frequent abnormalities associated with MDS [4,5]. While often associated with a good prognosis and infrequently with transformation to leukemia, our patient was noted to have a phenotype three months prior consistent with less than 5% blasts. On her repeat phenotyping two weeks prior to presentation, her flow cytometry showed an increase in CD34+ myeloblasts to 8.2% in some areas, consistent with MDS with excess blasts, type 1. This type is known to progress to acute myeloid leukemia. FISH analysis was significant for monosomy in chromosome 7. Monosomy 7 is a recurrent, non-random abnormality commonly observed in MDS and AML. In MDS, it is known to be associated with high-risk progression to AML and less favorable prognosis. Rearrangements in the short arm of chromosome 12 (in particular 12p13) are seen in both myeloid and lymphoid disorders. However, isochromosome 18q results in the loss of the short arm (18p) and duplication in the long arm (18q) [6]. This is a rare aberration found in a large spectrum of hematologic malignancies, often in older patients with complex karyotypes and largely in lymphoid malignancies. Unfortunately, while our patient was set to begin outpatient treatment, she developed an infectious process, possibly secondary to neutropenia, which altered the initial interpretation of her complete blood count. Her initial leukocytosis was attributed to an infectious process, which quickly transformed into a blast crisis. 

Sweet's syndrome (acute febrile neutrophilic dermatosis) is a rare reactive dermatosis that may be triggered by infections, medications, systemic diseases, or hematologic malignancies. Sweet's syndrome was initially described by Robert Douglas Sweet in the mid-1960s. At that time, he described raised, painful plaques on the extremities and necks of middle-aged females which he attributed to an immune reaction. The first association of Sweet's syndrome with malignancy was described in the 1990s and subsequent reports have confirmed associations with hematological malignancies, specifically acute myeloid leukemia [7]. In 2015, Kulasekararaj et al. studied 31 patients with Sweet's syndrome and demonstrated that T-helper 17 cells (Th17) were increased in low-risk MDS which correlated with increased apoptosis of the bone marrow and elevated serum levels of pro-inflammatory cytokines. These findings suggested an immunological phase of the disease before its transformation into high-risk leukemia. Our patient exhibited features of relapsing, recurrent Sweet's syndrome which coincided with malignant transformation to acute leukemia. Because her initial presentation included infectious diarrhea, her colchicine was held which further exacerbated her recurrence. While the biopsy of her abdominal lesions proved to be negative for Sweet's syndrome, the overall impression of fever, pneumonitis, and central nervous system (CNS) involvement was clinically worrisome and the patient was treated with systemic corticosteroids. 

The International Prognostic Scoring System (IPSS) (Table 1) is used by clinicians to assign a risk score and risk group. Each prognostic factor equates to a particular severity score. The summation of these values leads to an overall risk score which classifies patients based on their likelihood of progression to acute leukemia. The IPSS is the most commonly used method for prognostication in myelodysplastic syndrome and utilizes three key pathologic characteristics: (1) the percentage of leukemic blast cells in the marrow, (2) the type of chromosomal changes, if any, in the marrow cells (i.e., the cytogenetics), and (3) the presence of one or more cytopenias. The revised IPSS (IPSS-R) (Table 2) covers these same factors but identifies them in more detail with each cytopenia given an individual category and the inclusion of more cytogenetics. Upon presentation, our patient’s IPSS and IPSS-R scores were 1 (very low; 8% blasts, good karyotype, 2-3 cytopenias) and 5.5 (high risk, 8% blasts, good karyotype, hemoglobin less than 8, platelets less than 50, and absolute neutrophil count >0.8), respectively. 

**Table 1:** International Prognostic Scoring System (IPSS)

Prognostic Factor	Category	Score
Percentage of blast cells in the bone marrow		
	Less than 5%	0 points
	5% to 10%	0.5 point
	11% to 20%	1.5 points
	21% to 30%	2.0 points
Cytogenetics (chromosomal changes)		
	None or del(5q) or del (20q)	0 points
	Three or more abnormalities or an abnormal chromosome 7	1 point
	Other abnormalities	0.5 point
Number of cytopenias		
	None or one	0 points
	Two or three	0.5 point
Key: del = deletion		

**Table 2:** International Prognostic Scoring System - Revised (IPSS-R)

Prognostic Factor	Category	Score
Percentage of blast cells in the bone marrow		
	Less than or equal to 2%	0 points
	Greater than 2%, but less than 5%	1 point
	Between 5% to 10%	2 points
	Greater than 10%	3 points
Cytogenetics (chromosomal changes)		
	-Y or del(11q)	0 points
	Normal, del(5q), del(12p), del(20q), double + del(5q)*	1 point
	del(7q), +8, +19, i(17q), any other single or double independent clone**	2 points
	-7, inv(3), +(3q), del(3q), double deletion in 7 or del(7q), or 3 abnormalities	3 points
	More than 3 abnormalities	4 points
Hemoglobin concentration (g/dL)		
	Equal to or greater than 10	0 points
	Between 8 and 10	0.5 point
	Less than 8	1.5 points
Platelet count (x10^9^/L)		
	Equal to or greater than 100	0 points
	Between 50 and 100	0.5 point
	Less than 50	1 point
Absolute neutrophil count [(ANC) x 10^9^/L)		
	Equal to or greater than 0.8	0 points
	Less than 0.8	0.5 point
Risk Group Based on Total Risk Score		
	1.5 or less	Very Low
	2 to 3	Low
	3.5 to 4.5	Intermediate
	5 to 6	High
	6.5 or more	Very High
Key: del = deletion *double + del(5q) is a deletion in 5 q with another cytogenetic abnormality **a single clone may have many abnormalities, all of them occurring simultaneously in the same cell		

The International Prognostic Scoring System can be used to predict survival and leukemic transformation in patients with myelodysplastic syndrome. Approximately 67% of patients with MDS have lower risk disease at diagnosis with favorable prognosis. Based on her IPSS-R score, the median survival time for patients in this risk group is estimated to be 1.6 years and 25% of patients transform to AML. Dayanni et al [8] studied low-risk MDS patients characterized by IPSS to determine the most common cause of death. In this retrospective review, 9% of patients had a deletion in 5q and the most common causes of death were from infection (38%), AML transformation (15%), and hemorrhage (13%).

## Conclusions

The majority of patients with low- or intermediate-risk MDS die from complications related to their malignant condition. Approximately half of patients develop infections or hemorrhage as a complication of pancytopenia. Progression from a low/intermediate-risk disease to a phenotype that has a higher risk of malignant transformation may cause rapid patient decline. Progression from MDS to AML is a complex process involving multiple clonal genetic abnormalities. Patients with monosomy 7 are known to have a higher risk of progression to AML, and when coupled with rare co-mutations such isochromosome 18 often have poor prognosis. Patients with MDS who present with infections should be promptly evaluated for progression of their disease as prompt hematologic consultation may provide vital early assistance with the decision for anti-infectives versus cytoreductive therapies. Complications from leukemic transformation, including leukostasis and Sweet's syndrome, should be adequately and promptly addressed as neutropenia and pro-inflammatory conditions may lead to rapid mortality.
